# Supplementary material for: Landscapes and bacterial signatures of mucosa-associated intestinal microbiota in Chilean and Spanish patients with inflammatory bowel disease
Source: Microb Cell. 2021 Jun 18;8(9):223–38. doi: 10.15698/mic2021.09.760 (PMC8404152; doi:10.15698/mic2021.09.760)
Supplement: Supplementary file 1 [file mic-08-223-s01.pdf]

Supplementary Information

Landscapes and bacterial signatures of mucosa-associated intestinal microbiota in Chilean and Spanish patients with inflammatory bowel disease

Nayaret Chamorro[1*], David A. Montero[1,2*], Pablo Gallardo[3], Mauricio Farfán[3], Mauricio Contreras[4], , Marjorie De la Fuente[2], Karen Dubois[2], Marcela A. Hermoso[2], Rodrigo Quera[5,6], Marjorie Pizarro-Guajardo[7,8,9], Daniel Paredes-Sabja[7,8,9], Daniel Ginard[10], Ramon Rosselló-Móra[11], Roberto Vidal[1,8,12 **]

[1] Programa de Microbiología y Micología, Instituto de Ciencias Biomédicas, Facultad de Medicina, Universidad de Chile, Chile

[2] Programa de Inmunología, Instituto de Ciencias Biomédicas, Facultad de Medicina, Universidad de Chile, Chile.

[3] Facultad de Medicina, Departamento de Pediatría y Cirugía Infantil, Campus Oriente-Hospital Dr. Luis Calvo Mackenna, Universidad de Chile, Chile.

[4] Facultad de Ciencias, Departamento de Física, Universidad Metropolitana de Ciencias de la Educación, Santiago, Chile.

[5] Programa Enfermedad Inflamatoria Intestinal. Servicio de Gastroenterología, Clínica Las Condes, Santiago, Chile

[6] Gastroenterología, Clínica Universidad de Los Andes, Santiago, Chile

[7] Microbiota-Host Interactions and Clostridia Research Group, Departamento de Ciencias Biológicas, Facultad de Ciencias de la Vida, Universidad Andrés Bello, Santiago, Chile.

[8] ANID - Millennium Science Initiative Program - Millennium Nucleus in the Biology of Intestinal Microbiota, Santiago, Chile.

[9] Department of Biology, Texas A&M University, College Station, TX, 77843, USA

[10] Department of Gastroenterology and Palma Health Research Institute, Hospital Universitario Son Espases, Palma de Mallorca, Spain.

[11] Grupo de Microbiología Marina, IMEDEA (CSIC-UIB), Esporles, Illes Balears, Spain

[12] Instituto Milenio de Inmunología e Inmunoterapia, Facultad de Medicina, Universidad de Chile, Chile

* These authors contributed equally. ** Corresponding author. Correspondence should be addressed to Roberto Vidal (email: rvidal@uchile.cl)

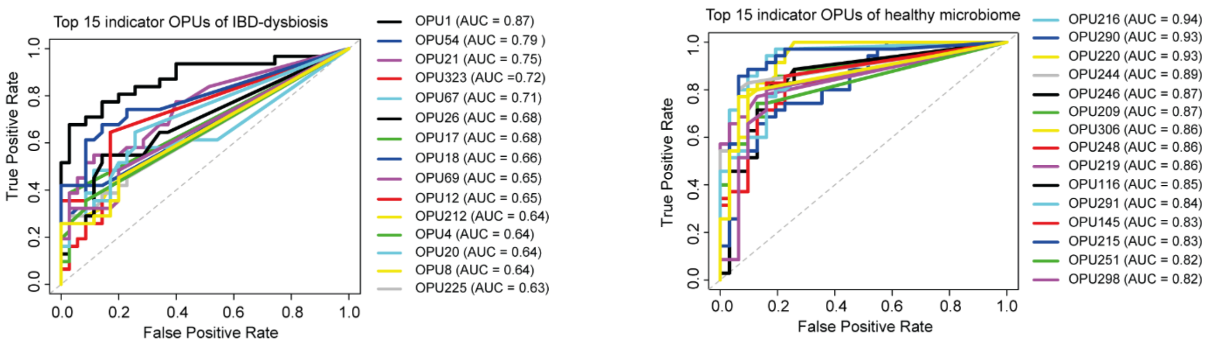

SUPPLEMENTARY FIGURE 1. Evaluation of OPUs as diagnostic biomarkers in IBD. OPUs identified by the LEfSe algorithm as differentially abundant in dysbiotic IBD patients or controls were also evaluated as biomarkers to discriminate between dysbiosis and eubiosis. The receiver operating characteristics (ROC) analysis was performed using the easyROC server [1]. AUC, Area under the curve.

REFERENCES

1. Goksuluk D, Korkmaz S, Zararsiz G, and Karaagaoglu AE (**2016**). EasyROC: An interactive web-tool for roc curve analysis using r language environment. **R J**. 8(2): 213–230. doi: 10.32614/rj-2016-042.
